# Age- and Sex-Associated Impacts of Body Mass Index on Stroke Type Risk: A 27-Year Prospective Cohort Study in a Low-Income Population in China

**DOI:** 10.3389/fneur.2019.00456

**Published:** 2019-05-01

**Authors:** Hongfei Gu, Shuang Shao, Jie Liu, Zhenqian Fan, Yu Chen, Jingxian Ni, Conglin Wang, Jun Tu, Xianjia Ning, Yongzhong Lou, Bin Li, Jinghua Wang

**Affiliations:** ^1^Department of Neurology, Tianjin Haibin People's Hospital, Tianjin, China; ^2^Department of Endocrinology and Metabolism, The Second Hospital of Tianjin Medical University, Tianjin, China; ^3^Department of Neurology, Tianjin Medical University General Hospital, Tianjin, China; ^4^Laboratory of Epidemiology, Tianjin Neurological Institute, Tianjin, China; ^5^Key Laboratory of Post-Neuroinjury Neuro-Repair and Regeneration in Central Nervous System, Tianjin Neurological Institute, Ministry of Education, Tianjin, China; ^6^Department of Geriatrics, Tianjin Medical University General Hospital, Tianjin, China

**Keywords:** stroke, BMI, subtype, epidemiology, risk factors

## Abstract

The relationship between body mass index (BMI) and stroke type has remained controversial despite studies demonstrating that BMI is related to stroke risk, especially in specific groups. We assessed the age- and sex-associated impacts of BMI on stroke type in a low-income, poorly educated population in China. The association of BMI with stroke type was estimated using Cox regression analyses in this prospective cohort study, after adjusting for sex, age, education level, hypertension, diabetes, smoking, and alcohol drinking status. During the follow-up period, 638 stroke cases occurred among the 3,906 participants included in this prospective study. For men aged <65 years, being overweight was an independent predictor of all stroke subtypes, compared with normal-weight individuals; the hazard ratios (HRs) and 95% confidence intervals (CIs) were 1.98 (1.52–2.58) for total stroke, 1.69 (1.22–2.33) for ischemic stroke, and 3.62 (2.09–6.25) for hemorrhagic stroke, all *P* < 0.001. Being underweight was also an independent predictor of hemorrhagic stroke (HR, 5.10; 95%CI, 1.80–14.50, *P* = 0.002). For women <65-years-old, being overweight was a risk factor for total (HR, 1.38; 95% CI, 1.01–1.89; *P* = 0.044) and hemorrhagic strokes (HR, 2.06; 95% CI, 1.00–4.28; *P* = 0.050); obesity was a risk factor for total (HR, 2.47; 95% CI, 1.60–3.82) and ischemic strokes (HR, 2.53; 95% CI, 1.54–4.15), all *P* < 0.001. These findings suggest that weight management should be a high priority for substantially reducing the heavy burden of strokes in rural China among both men and women <65-years-old; men<65-years-old should maintain their weight within a reasonable range.

## Introduction

Stroke has recently become the leading cause of death in China (1), accounting for almost one-third of the total number of stroke-related deaths, worldwide (2). In 2013, 27 of China's 33 provinces reported strokes as the main cause of death (3). The proportion of adults with a body mass index (BMI) ≥25 kg/m^2^ increased globally between 1980 and 2013, among both men (from 28.8 to 36.9%) and women (from 29.8 to 38.0%) (4).

The positive association between BMI and ischemic stroke is well-established (5–8). For hemorrhagic stroke, the results of previous studies have been more disparate, with some reports showing a linear, positive correlation (6, 7, 9–11) with BMI, others showing a J-shaped or U-shaped relationship (9, 12, 13), and some showing no correlation (14, 15) or a linearly opposite correlation (16). Such discrepancies may be due to differences in the risk factors and stroke incidences among different populations.

In low- and middle-income countries, 22.4% of the stroke burden (measured using disability-adjusted life years) is attributable to high BMIs; separate estimates for pathological types and patient age and sex were not reported (17). Moreover, our previous study revealed the increased burden of stroke in young and middle-aged adults (18). Thus, we aimed to assess the age-and sex associated relationship between BMI and total, ischemic, and hemorrhagic strokes, in a rural population in Tianjin China.

## Methods

### Study Population and Sample Process

The study cohort was derived from a population-based stroke monitoring study. The baseline investigation was performed in October 1991, and stroke events were recorded until to February 2018. The study population was previously described (18–21). Briefly, it included 14,936 people (in 1991), 95% of whom were low-income farmers living in 18 administrative villages. The main source of income for the residents was cereal crop production, with annual per capita incomes of <100 USD in 1990 and <1,000 USD in 2010 (22).

The sampling method used in this cohort study was also previously reported (23). Briefly, all residents within the 18 administrative villages in the region were recruited into the study. The villages were divided into three geographic regions (east, south, and north) and two villages from each region were randomly selected. Using a stratified cluster sampling approach, we selected all residents who were from the six villages, aged ≥15 years, and without evidence of coronary heart disease or stroke to participate in the study. For the purposes of this analysis, only participants aged ≥18 years were included.

The investigative protocol was approved by the ethics committee of Tianjin Medical University General Hospital, and a written informed consent was obtained from each participant.

### Baseline Information

Demographic characteristics (including sex, age, and educational level), past medical history (including hypertension, diabetes, stroke, and cardiovascular disease), and personal lifestyle (including smoking habits and alcohol consumption) were recorded, and these were from self-reported by participants. All information was collected in face-to-face interviews by trained local researchers conducting. Physical examinations, including blood pressure (BP), height, and weight measurements, were also conducted during the interviews.

### Measurement Methods and Definition

BP was measured in the supine position using methods described previously (23). Height and weight were measured using a height and weight meter with the participants in light clothing. Body mass index (BMI) was calculated as weight (kg) divided by the square of height (m).

Hypertension was defined as a self-reported previous hypertension history, or use of antihypertensive medicine, or SBP/DBP of 140/90 mmHg at baseline; diabetes was defined as a self-reported previous disease history or current use of antidiabetics. Underweight was defined as a BMI<18 kg/m^2^, normal weight as a BMI of 18–23.9 kg/m^2^, overweight as a BMI of 24–27.9 kg/m^2^, and obesity as a BMI ≥28 kg/m^2^ (24).

### Case Definition

We previously published the stroke monitoring and protocol details used to diagnose stroke and determine stroke types (18–21). Stroke was defined as a first-ever, acute, focal, neurological deficit of vascular etiology that lasted >24 h.

All first-ever stroke events were diagnosed as full clinical stroke, with obvious clinical signs and symptoms. Stroke events including hemorrhagic, ischemic, and undetermined strokes, were diagnosed by neuroimaging within 72 h of stroke onset. Hemorrhagic stroke was defined as an intracerebral hemorrhage (ICH) or subarachnoid hemorrhage. Ischemic stroke (IS) was defined as a thrombotic brain infarction, cardioembolic stroke, or lacunar infarct. Undetermined stroke was defined as stroke that could not be classified into the previously described types because there was absence the neuroimaging evidence; but all these patients were symptomatic stroke.

In this study, subarachnoid hemorrhage, transient ischemic attacks (TIAs) and silent strokes (diagnosed via imaging, only) were excluded. Patients with transient symptoms but with neuroimaging evidence of cerebral infarctions were considered stroke cases (25). Moreover, those patients caused by other causes with stroke-relative symptom were excluded in this study.

Subgroup analysis of stroke types in this study was included IS, ICH, and stroke (included IS, ICH, and undetermined stroke) to assess the association of BMI with stroke risk.

### Stroke Event Ascertainment and Processes

The stroke case ascertainment process was as described previously (18). Briefly, stroke events were reported according to predefined procedures. First, a local licensed village doctor reported stroke events to physicians in the community hospital within 24 h of patient presentation. Second, community hospital physicians visited surviving patients within 72 h to obtain clinical feature information and confirm the occurrence of stroke. Each month, these physicians reported confirmed stroke events (diagnosed by imaging) to Tianjin Medical University General Hospital (TMUGH); suspected events (no imaging performed) were also reported in a timely manner. Finally, as soon as possible, a TMUGH neurologist identified suspected cases during door-to-door interviews.

The community hospital physicians and TMUGH neurologist collected information regarding stroke onset during interviews with the patient or patient's family. The collected information included patient demographics, time of stroke onset, clinical signs, and previous stroke status. The TMUGH neurologist also obtained other information, including therapy and post-discharge outcomes, the during interviews with the surviving patients or their family members.

### Statistical Analysis

Continuous variables (age, BP, and BMI) are expressed as means (standard deviations) and categorical variables are expressed as 95% confidence intervals (CIs). Age-standardized incidences were calculated using the direct method of world standard population age groupings: <45, 45–64, and ≥65 years (26). Subgroup analyses were conducted to evaluate the risk of first-ever stroke by age (<65 years, and ≥65 years), educational attainment (illiterate, 1–6 years of schooling, and >7 years of schooling), BMI group (underweight, normal, overweight, obese), smoking status (never smoked, ever smoked, and current smoking), and alcohol consumption status (never drank, ever drank, and current drinking). Association of BMI with the risk of developing stroke was assessed by Kaplan-Meier survival analysis and presented as a survival curve. We estimated the hazard ratio (HR) of BMI on stroke, with and without adjusting for sex, age, education level, hypertension, diabetes, smoking, and alcohol drinking status, using Cox regression analyses. The follow-up time (recorded in years) was calculated as the interval between the date at baseline and the date of the occurring stroke for patients experiencing a first-ever stroke during the study period. For participants without further stroke events, the follow-up time was defined as 26.3 years. Moreover, for participants who died during the study periods, the follow-up time was defined as the interval between baseline and the date of death. Data for patients who were lost to follow-up or who emigrating were censored. All statistical analyses were performed using SPSS for Windows (version15.0; SPSS, Chicago, IL, USA); *P*-values < 0.05 were considered statistically significant.

## Results

### Participant Characteristics

During the 27 years of follow-up, a total of 638 strokes occurred among the 3,906 participants, including 404 ischemic, 121 hemorrhagic, and 113 undefined strokes. The cumulated incidence of stroke in this population was 16.3% overall.

The 3,906 individuals involved in the study had an average age of 41.74 years and included 1,834 men (47.0%) and 2,072 women (53.0%); 40.7% of the participants had never received any formal education (men, 36.8%; women, 44.1%). The prevalence of hypertension and diabetes at baseline was 31.0 and 0.1%, respectively; the percentages of participants who were current smokers and alcohol consumers were 25.7 and 15.4%, respectively. The average BMI for these individuals was 22.58 kg/m^2^ at baseline (Table 1).

**Table 1:** Demographic characteristics and risk factors of all participants in this study.

**Risk factors**	**Total**	**Men**	**Women**	***P***
Total, n (%)	3,906	1,834 (47.0)	2,072 (53.0)	
Age, means (*SD*), years				
	41.74 (16.58)	42.51 (16.89)	41.07 (16.28)	0.007
Age group, *n* (%)				0.009
<65 years	3,424 (87.7)	1,581 (86.2)	1,843 (88.9)	
≥65 years	482 (12.3)	253 (13.8)	229 (11.1)	
Education, *n* (%)				<0.001
0 years	1,588 (40.7)	675 (36.8)	913 (44.0)	
1~6 years	978 (25.0)	508 (27.7)	470 (22.7)	
≥7 years	1,340 (34.3)	651 (35.5)	689 (33.3)	
Baseline Hypertension, *n* (%)				0.286
No	2,695 (69.0)	1,250 (68.2)	1,445 (69.7)	
Yes	1,211 (31.0)	584 (31.8)	627 (30.3)	
Baseline Diabetes*, n* (%)				0.060
No	3,902 (99.9)	1,834 (100)	2,068 (99.8)	
Yes	4 (0.1)	0	4 (0.2)	
Smoking status, *n* (%)				<0.001
Current smoking	1,002 (25.7)	921 (50.2)	81 (3.9)	
Ever smoking	112 (2.9)	101 (5.5)	11 (0.5)	
Never smoking	2,792 (71.5)	812 (44.3)	1,980 (95.6)	
Alcohol consumption, *n* (%)				<0.001
Current drinking	602 (15.4)	577 (31.5)	25 (1.2)	
Ever drinking	16 (0.4)	16 (0.9)	0	
Never drinking	3,288 (84.2)	1,241 (67.6)	2,047 (98.8)	
BMI, means (*SD*), Kg/m^2^				
	22.58 (2.80)	22.32 (2.37)	22.83 (3.11)	<0.001
BMI group, *n* (%)				<0.001
Underweight	175 (4.5)	62 (3.4)	113 (5.5)	
Normal weight	2,722 (69.7)	1,404 (76.6)	1,318 (63.6)	
Overweight	847 (21.7)	332 (18.1)	515 (24.9)	
Obesity	162 (4.1)	36 (2.0)	126 (6.1)	
SBP, means (*SD*), mmHg				
	127.39 (20.27)	127.92 (17.57)	126.92 (22.39)	0.120
DBP, means (*SD*), mmHg				
	79.95 (11.41)	80.60 (10.59)	79.37 (12.06)	0.001

### Distribution of Stroke Risk Factors in This Population at Baseline by BMI

The prevalence of overweight and obesity was much higher among women than among men (24.9 vs. 18.1%, and 6.1 vs. 2.0%, respectively; *P* < 0.001). In this population, participants with hypertension tended to have higher rates of being overweight or obese than those without hypertension, with the corresponding rates of overweight and obesity being 28.5 vs. 18.6%, and 7.3 vs. 2.7%, respectively; *P* < 0.001 (Table 2).

**Table 2:** Distribution of stroke risk factors in this population at baseline by BMI.

**Risk factors**	**Underweight**	**Normal**	**Overweight**	**Obesity**	***P***
Gender, *n* (%)					<0.001
Male	62 (3.4)	1,404 (76.6)	332 (18.1)	36 (2.0)	
Female	113 (5.5)	1,318 (63.6)	515 (24.9)	126 (6.1)	
Age group, *n* (%)					<0.001
<65 years	125 (3.7)	2,388(69.7)	763 (22.3)	148 (4.3)	
≥65 years	50 (10.4)	334 (69.3)	84 (17.4)	14 (2.9)	
Education, *n* (%)					0.031
0 years	87 (5.5)	1,092 (68.8)	342 (21.5)	67 (4.2)	
1~6 years	44 (4.5)	661 (67.6)	225 (23.0)	48 (4.9)	
≥7 years	44 (3.3)	969 (72.3)	280 (20.9)	47 (3.5)	
Smoking, *n* (%)					<0.001
Current smoking	39 (3.9)	758 (75.6)	179 (17.9)	26 (2.6)	
Ever smoking	4 (3.6)	76 (67.9)	28 (25.0)	4 (3.6)	
Never smoking	132 (4.7)	1,888 (67.6)	640 (22.9)	132 (4.7)	
Alcohol, *n* (%)					0.057
Current drinking	17 (2.8)	439 (72.9)	130 (21.6)	16 (2.7)	
Ever drinking	0	14 (87.5)	2 (12.5)	0	
Never drinking	158 (4.8)	2,269 (69.0)	715 (21.7)	146 (4.4)	
Hypertension, *n* (%)					<0.001
No	122 (4.5)	1,997 (74.1)	502 (18.6)	74 (2.7)	
Yes	53 (4.4)	725 (59.9)	345 (28.5)	88 (7.3)	
Diabetes, *n* (%)					0.028
No	174 (4.5)	2,721 (69.7)	846 (21.7)	161 (4.1)	
Yes	1 (25.0)	1 (25.0)	1 (25.0)	1 (25.0)	

### Association of BMI With Total, Ischemic, and Hemorrhagic Stroke in Kaplan-Meier Survival Analysis

**Figure 1:**
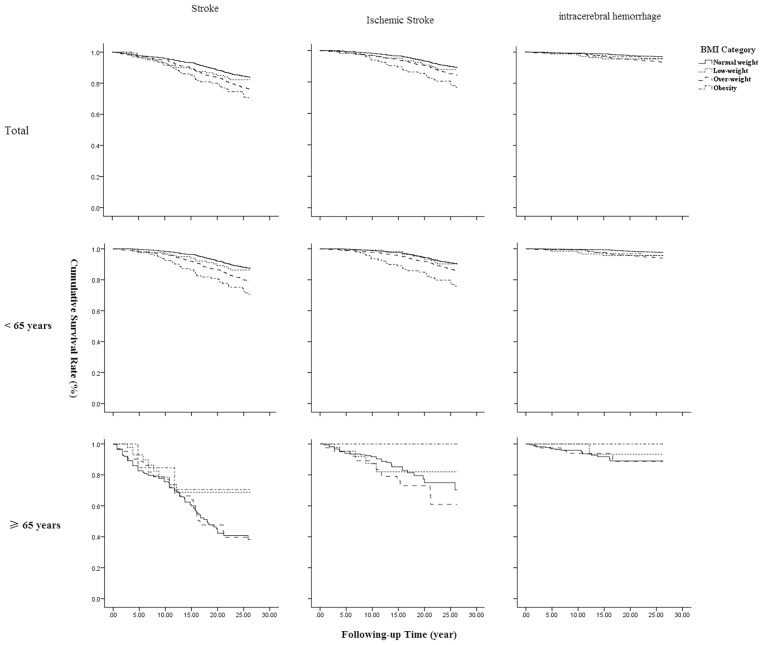
Association of BMI with stroke survival by types and age.

Figure 1 shows that BMI is associated with occurrence of a first-ever stroke overall, all *P* < 0.001. The highest survival rate was observed among patients with obesity at baseline across all stroke types. Similar results were found in patients aged <65 years. However, a significant association was not found in elderly patients aged 65 years and older.

### Association of BMI With Total, Ischemic, and Hemorrhagic Stroke Using Cox Regression Analysis

**Figure 2:**
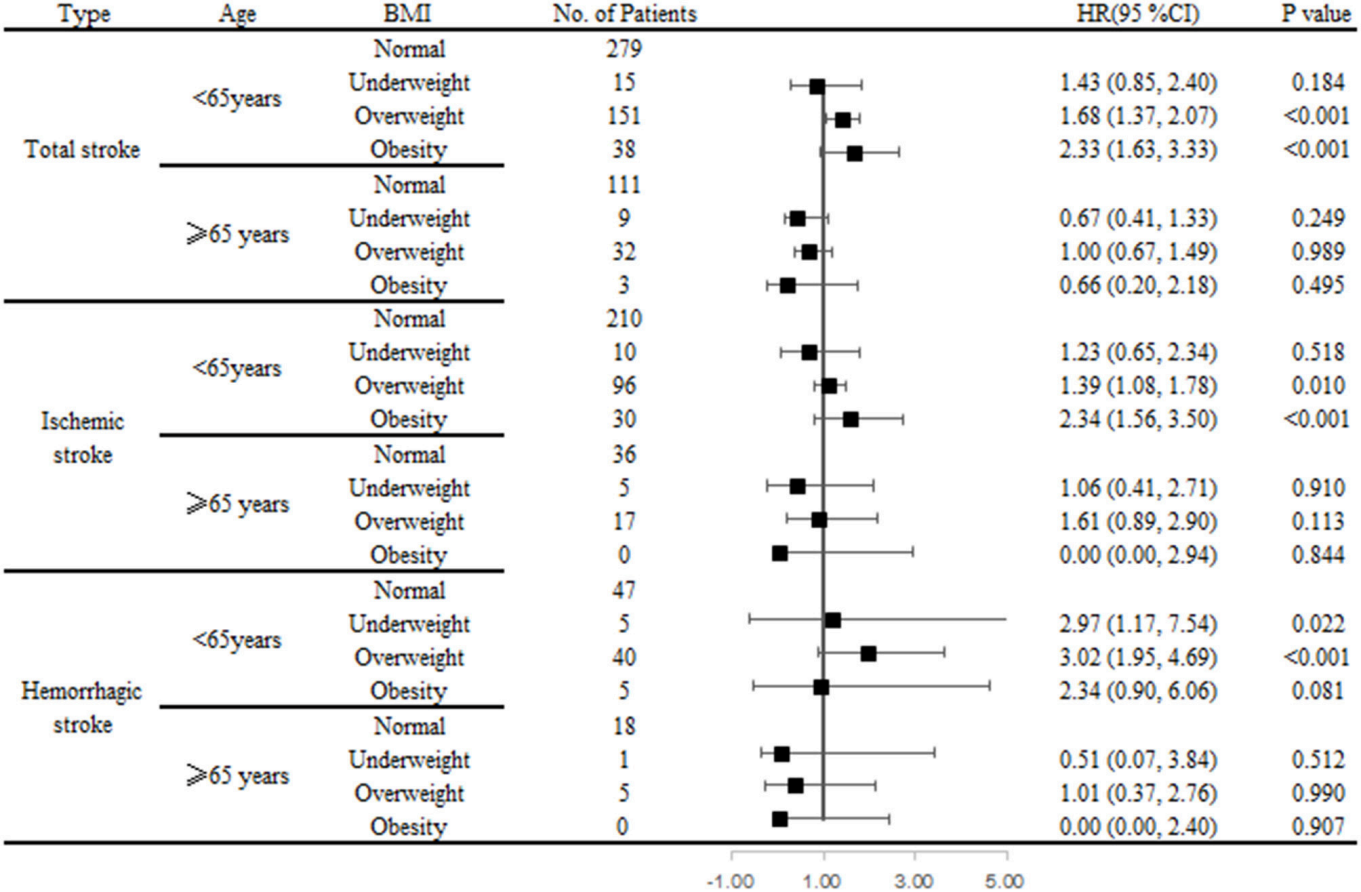
Association of BMI and stroke risk by types and age groups using Cox regression analysis.

Compared with patients of normal baseline weights, the HRs (95% CIs) associated with being overweight at baseline were 1.48 (1.24–1.77; *P* < 0.001) for total stroke, 1.43 (1.14–1.80; *P* = 0.002) for ischemic stroke, and 2.34 (1.58–3.47; *P* < 0.001) for hemorrhagic stroke, after adjusting for confounders. Obesity at baseline was significantly and positively associated with both total and ischemic stroke, with HRs (95% CIs) compared to normal-weight individuals, of 2.00 (1.44–2.79) for total stroke and 2.16 (1.46–3.21) for ischemic stroke, respectively, all *P* < 0.001. There was no statistically significant association between being underweight at baseline and stroke (Table 3; Figure 2).

**Table 3:** Association of BMI with total, ischemic, and hemorrhagic stroke using Cox regression analysis.

**Subtypes**	**Underweight**	**Normal**	**Overweight**	**Obesity**
**Total stroke**				
Case (*n* = 638)	24	390	183	41
Adjusted HRs (95%CI)	1.06 (0.70, 1.61)	1.0	1.46 (1.22, 1.75)*	2.00 (1.44, 2.79)*
**Ischemic stroke**				
Case (*n* = 404)	15	246	113	30
Adjusted HRs (95%CI)	1.22 (0.72, 2.06)	1.0	1.40 (1.11, 1.76)*	2.17 (1.46, 3.21)*
**Hemorrhagic stroke**				
Case (*n* = 121)	6	65	45	5
Adjusted HRs (95%CI)	1.81 (0.77, 4.22)	1.0	2.41 (1.63, 3.57)*	1.80 (0.71, 4.59)

* *P < 0.05*.

### Association of BMI With Total, Ischemic, and Hemorrhagic Stroke by Age Using Cox Regression Analysis

Among individuals aged <65 years, being overweight was an independent determinant of stroke, regardless of stroke type, compared with normal weight individuals; the HRs (95% CIs) were 1.69 (1.38–2.07; *P* < 0.001) for total stroke, 1.42 (1.11–1.81; *P* = 0.006) for ischemic stroke, and 2.93 (1.88–4.56; *P* < 0.001) for hemorrhagic stroke. Obesity was significantly associated with developing both total (HR, 2.35; 95% CI, 1.65–3.36; *P* < 0.001) and ischemic (HR, 2.35; 95% CI, 1.57–3.51; *P* < 0.001) strokes. Being underweight was also associated with hemorrhagic stroke risk (HR, 3.18; 95% CI, 1.26–8.05; *P* = 0.002). However, among individuals aged ≥65 years, there was no correlation between BMI and stroke, regardless of type (Table 4).

**Table 4:** Association of BMI with total, ischemic, and hemorrhagic stroke by age using Cox regression analysis.

**Subtypes**	** <65 years**				**≥65 years**			
	**Underweight**	**Normal**	**Overweight**	**Obesity**	**Underweight**	**Normal**	**Overweight**	**Obesity**
**Total stroke**								
Case (*n* = 638)	15	279	151	38	9	111	32	3
Adjusted HR (95%CI)	1.43 (0.85, 2.40)	1.0	1.68 (1.37, 2.07)^*^	2.33 (1.63, 3.33)^*^	0.67 (0.34, 1.33)	1.0	1.00 (0.67, 1.49)	0.66 (0.20, 2.18)
*P*-value	0.184		<0.001	<0.001	0.249		0.989	0.495
**Ischemic stroke**								
Case (*n* = 404)	10	210	96	30	5	36	17	0
Adjusted HR (95%CI)	1.23 (0.65, 2.34)	1.0	1.39 (1.08, 1.78)^*^	2.34 (1.56, 3.50)^*^	1.06 (0.41, 2.71)	1.0	1.61 (0.89, 2.90)	0.00 (0.00, 2.94)
*P*-value	0.518		0.010	<0.001	0.910		0.113	0.844
**Hemorrhagic stroke**								
Case (*n* = 121)	5	47	40	5	1	18	5	0
Adjusted HR (95%CI)	2.97 (1.17, 7.54)^*^	1.0	3.02 (1.95, 4.69)^*^	2.34 (0.90, 6.06)	0.51 (0.67, 3.8)	1.0	1.01 (0.37, 2.76)	0.00 (0.00, 2.40)
*P*-value	0.022		<0.001	0.081	0.512		0.990	0.907

* *P < 0.05*.

### Association of BMI With Total, Ischemic, and Hemorrhagic Stroke Using Cox Regression Analysis Among Population Aged <65 Years

For men aged <65 years, being overweight was an independent determinant of all stroke types, relative to normal weight individuals; the HRs (95% CIs) were 1.98 (1.52–2.58) for total stroke, 1.69 (1.22–2.33) for ischemic stroke, and 3.62 (2.09–6.25) for hemorrhagic stroke (all *P* < 0.001). Importantly, being underweight was also an independent determinant of hemorrhagic stroke (HR, 5.10; 95%CI, 1.80–14.50, *P* = 0.002). However, there was no relationship between obesity and any stroke type.

For women aged <65 years, compared with normal weight individuals, being overweight was a risk factor for total (HR, 1.38; 95% CI, 1.01–1.89, *P* = 0.044) and hemorrhagic (HR, 2.06; 95% CI, 1.00–4.28, *P* = 0.050) strokes; obesity was a risk factor for total (HR, 2.47; 95% CI, 1.60–3.82) and ischemic (HR, 2.53; 95%CI, 1.54–4.15) stroke, all *P* < 0.001; being underweight was not associated with any stroke type (Table 5).

**Table 5:** Association of BMI with total, ischemic, and hemorrhagic stroke using Cox regression analysis among population aged <65 years.

	**Men**				**Women**			
	**Underweight**	**Normal**	**Overweight**	**Obesity**	**Underweight**	**Normal**	**Overweight**	**Obesity**
**Total stroke**								
Case (*n* = 483)	7	180	83	10	8	99	68	28
AdjustedHR (95%CI)	1.41 (0.66, 3.02)	1.0	1.92 (1.47, 2.51)^*^	1.91 (1.00, 3.66)	1.40 (0.67, 2.89)	1.0	1.44 (1.04, 1.96)^*^	2.54 (1.64, 3.94)^*^
**Ischemic stroke**								
Case (*n* = 346)	3	132	53	8	7	78	43	22
Adjusted HR (95%CI)	0.82 (0.26, 2.59)	1.0	1.60 (1.15, 2.22)^*^	1.96 (0.95, 4.06)	1.58 (0.72, 3.45)	1.0	1.16 (0.79, 1.69)	2.62 (1.59, 4.32)^*^
**Hemorrhagic stroke**								
Case (*n* = 97)	4	32	24	2	1	15	16	3
Adjusted HR (95%CI)	4.56 (1.59, 13.08)^*^	1.0	3.61 (2.09, 6.24)^*^	2.79 (0.65, 12.02)	1.07 (0.14, 8.18)	1.0	2.21 (1.08, 4.53)^*^	1.70 (0.48, 6.01)

* *P < 0.05*.

## Discussion

In this large, prospective, cohort study of 3906 persons with 27 years of follow-up, we evaluated the age- and sex-associated correlations of BMI with the risk of total, ischemic, and hemorrhagic strokes. Overall, being overweight at baseline increased the risk of both ischemic and hemorrhagic strokes; obesity increased the risk of ischemic stroke. Additionally, the positive association of BMI with stroke risk was only observed in participants aged <65 years and was different in men compared to women. In this age group, being overweight increased the risk of both ischemic and hemorrhagic strokes, while being underweight also increased the risk of hemorrhagic stroke, in men. Moreover, in women, being overweight increased the risk of hemorrhagic stroke and being obese increased the risk of total and ischemic strokes.

A meta-analysis involving 2 million participants from 10 countries (including countries in Asia, Europe, and North America) indicated that the relative risks for ischemic stroke were 1.22 for overweight individuals and 1.64 for obese individuals; hemorrhagic stroke was not associated with BMI (27). Previous studies in China showed that the incidences of total and ischemic strokes had a linear relationship with BMI and that there was no significant association between BMI and hemorrhagic stroke (8, 15). In the present study, being overweight or obese (compared to being of a normal weight) were risk factors for developing total and ischemic strokes for the overall population; an increased risk of hemorrhagic stroke was associated with being overweight. Interestingly, previous studies have indicated an increasing prevalence of hypertension, diabetes, and higher BPs in this population over the past decades (18–21, 23). These observations may partly explain the positive association of BMI with the risk of developing stroke in this rural population (28).

Several large prospective studies in eastern Asian individuals, aged <60 years, have failed to identify clear positive associations between the risk of hemorrhagic stroke and a BMI <25 kg/m^2^ (10, 12, 29). Among individuals with a BMI <25 kg/m^2^, the mean difference in SBP/DBP was at least 11/5 mmHg compared to normal weight individuals, corresponding to an HR of 1.6 for ICH (30). Further, the risk of hemorrhagic stroke increases with decreasing serum cholesterol levels (31). A study from the United States showed a positive association between elevated BMIs and stroke among individuals aged ≥65 years (32). Higher BMIs also raise the risk of stroke among men aged ≥70 years (33). However, unlike these previous studies, the present study showed that, among men aged <65 years, being overweight was a risk factor for total, ischemic, and hemorrhagic strokes, whereas being underweight was also associated with hemorrhagic stroke. For female participants aged <65 years, being overweight increased the risk of hemorrhagic stroke and being obese increased the risk of ischemic stroke. Among those aged ≥65 years, there were no correlations between BMI and the risk of total, ischemic, or hemorrhagic strokes. The lower prevalence of being overweight or obese among individuals aged ≥65 years compared with those aged <65 years, may have contributed to their lower stroke risk. Further, body fat becomes more likely to be located in the abdominal cavity as age increases; thus, BMI becomes a poorer indicator of overall and abdominal fatness in older individuals (34).

Previous studies in Western countries have shown that BMI is significantly associated with IS among middle-aged men and women (14, 16, 35). However, the association of BMI with hemorrhagic stroke remains controversial. One report showed a linearly opposing correlation (16) whereas others showed no correlation (14, 35). Studies from China indicate that being overweight or obese are risk factors for IS among middle-aged Chinese populations (11, 15); BMI was not associated with hemorrhagic stroke risk in men (15), but was positively correlated with that risk in women (11). Contrary to those observations, our findings indicate that being overweight increases the risk of both ischemic and hemorrhagic stroke and that being underweight also increases the risk of hemorrhagic stroke in men aged <65 years. Moreover, in women, being overweight increased the risk of hemorrhagic stroke and being obese increased the risk of IS. This may be related to the documented association between increased levels of inflammatory markers and the risk of IS (36, 37). Further, obesity is more strongly correlated with elevated high-sensitivity C-reactive protein levels in women than in men (38). Therefore, we hypothesize that metabolic changes related to obesity are more likely to affect the risk of stroke in women than in men. In addition, in the present study, both underweight and overweight individuals were at greater risk of hemorrhagic stroke among men aged <65 years, whereas only being overweight increased the risk of hemorrhagic stroke in women. Ultimately, larger studies are required to clarify the sex-specific effects of BMI on hemorrhagic stroke risk.

It is well known that increased adiposity has adverse effects on insulin sensitivity, lipid metabolism, autonomic tone, fibrinolysis, and inflammation, which contributes to endothelial dysfunction and atherosclerosis (39). In addition, obesity is associated with relatively higher levels of prothrombotic and inflammatory markers (e.g., plasminogen activator inhibitor-1 antigen, fibrinogen, and C-reactive protein) (40, 41), which are associated with IS (36, 42). These may explain the mechanism underlying the association of BMI with developing stroke.

There are several limitations to this study. First, the study population came from a township in northern China that is not representative of the overall national population. However, due to this study being a prospective cohort study in a low-income Chinese population, a larger population study design conducted over a longer period may have reduced the impact of limited representations on the results. Second, the study population was rural Chinese residents with low-incomes and low levels of education and this may limit generalization of the study's findings. However, such rural populations account for more than 50% of China's total population, so our results are representative of this part of the population. Third, the small numbers of patients experiencing stroke during the study periods may have decreased the statistical power to detect possible associations. Fourth, we did not collect all information regarding medicine use for all participants; however, due to their low socioeconomic status, the frequency of medicine use among this population is presumed to be quite low and is not expected to impact the validity of the results. In addition, no patients reported having dyslipidemia at baseline in this study. This may impact the integrated evaluation of the determinants of experiencing stroke. Finally, we only assessed BMI at baseline; central obesity and BMI variation were not addressed in this study. These reduced the power of comprehensively evaluating the association between obesity and stroke risk.

## Conclusions

Being overweight increased the risk of both ischemic and hemorrhagic strokes; obesity was only associated with an increased risk of IS. Additionally, the positive association between BMI and stroke risk was only observed in participants aged <65 years and the associations differed between men and women. Being overweight increased the risk of both ischemic and hemorrhagic strokes in men and being underweight increased their risk of hemorrhagic stroke. In women, being overweight increased the hemorrhagic stroke risk, whereas obesity increased their IS risks. The high prevalence of hypertension and elevated BP levels in this low-income population may partially explain the observed positive association between BMI and stroke risk. These findings suggest that weight management should be a high priority for substantially reducing the heavy burden of strokes in rural China, among both men and women aged <65 years; men should maintain their weight within a reasonable range.

## Ethics Statement

This study was carried out in accordance with the recommendations of the ethics committee of Tianjin Medical University General Hospital with written informed consent from all subjects. All subjects gave written informed consent in accordance with the Declaration of Helsinki.

## Author Contributions

JW, BL, and YL contributed to the conception and design of the work. HG, SS, JL, ZF, YC, JN, CW, and JT contributed the data acquisition. JW and XN contributed the analysis and interpretation of data for the work. HG and SS contributed drafting the work. JW and XN contributed revising the work for important intellectual content. All authors approved of the final version to be published, and agree to be accountable for all aspects of the work in ensuring that questions related to the accuracy or integrity of any part of the work are appropriately investigated and resolved.

### Conflict of Interest Statement

The authors declare that the research was conducted in the absence of any commercial or financial relationships that could be construed as a potential conflict of interest.
